# *Trichuris suis* induces human non-classical patrolling monocytes via the mannose receptor and PKC: implications for multiple sclerosis

**DOI:** 10.1186/s40478-015-0223-1

**Published:** 2015-07-25

**Authors:** Gijs Kooij, Rens Braster, Jasper J. Koning, Lisa C. Laan, Sandra J. van Vliet, Tamara Los, Anne Marieke Eveleens, Susanne M. A. van der Pol, Elisabeth Förster-Waldl, Kaan Boztug, Alexandre Belot, Katka Szilagyi, Timo K. van den Berg, Jaap D. van Buul, Marjolein van Egmond, Helga E. de Vries, Richard D. Cummings, Christine D. Dijkstra, Irma van Die

**Affiliations:** Department of Molecular Cell Biology and Immunology, Neuroscience Campus Amsterdam, VU University Medical Center, P.O. Box 7057, 1007 MB Amsterdam, The Netherlands; Divison of Neonatology, Paediatric Intensive Care and Neuropaediatrics, Department of Paediatrics and Adolescent Medicine, Medical University of Vienna, Vienna, Austria; CeMM Research Center for Molecular Medicine of the Austrian Academy of Sciences, Vienna, Austria; Hôpital Femme Mère Enfant, Hospices Civils de Lyon and Université de Lyon, Lyon, France; Department of Blood Cell Research, University of Amsterdam, Amsterdam, The Netherlands; Department of Molecular Cell Biology, Sanquin Research and Landsteiner Laboratory, Academic Medical Center, University of Amsterdam, Amsterdam, The Netherlands; Department of Biochemistry, Emory University School of Medicine, Atlanta, USA

**Keywords:** Auto-immunity, Multiple sclerosis, Innate immunity, *Trichuris suis*, Monocytes, Mannose receptor, Protein kinase C, PKCδ

## Abstract

**Introduction:**

The inverse correlation between prevalence of auto-immune disorders like the chronic neuro-inflammatory disease multiple sclerosis (MS) and the occurrence of helminth (worm) infections, suggests that the helminth-trained immune system is protective against auto-immunity. As monocytes are regarded as crucial players in the pathogenesis of auto-immune diseases, we explored the hypothesis that these innate effector cells are prime targets for helminths to exert their immunomodulatory effects.

**Results:**

Here we show that soluble products of the porcine nematode *Trichuris suis* (TsSP) are potent in changing the phenotype and function of human monocytes by skewing classical monocytes into anti-inflammatory patrolling cells, which exhibit reduced trans-endothelial migration capacity in an in vitro model of the blood–brain barrier. Mechanistically, we identified the mannose receptor as the TsSP-interacting monocyte receptor and we revealed that specific downstream signalling occurs via protein kinase C (PKC), and in particular PKCδ.

**Conclusion:**

This study provides comprehensive mechanistic insight into helminth-induced immunomodulation, which can be therapeutically exploited to combat various auto-immune disorders.

**Electronic supplementary material:**

The online version of this article (doi:10.1186/s40478-015-0223-1) contains supplementary material, which is available to authorized users.

## Introduction

Multiple sclerosis (MS) is a chronic inflammatory disease of the central nervous system in which autoreactive T cells and monocyte-derived macrophages cause severe brain tissue damage, leading to neurological deficits [1, 2]. Epidemiological studies have shown that there is an inverse correlation between the prevalence of MS and the occurrence of helminth (worm) infections [3]. Intriguingly, helminth-infected MS patients show significant lower number of relapses, reduced disability scores, and lower magnetic resonance imaging activity compared to uninfected MS subjects [4], suggesting a strong immunomodulatory effect of these helminths. Anthelmintic treatment of such patients was associated with significant increase in disease activity, as observed by both clinical and radiological observations [4]. These data indicate that probiotic helminth administration may be a promising avenue to treat MS. Indeed, recent clinical tests in MS using the porcine nematode *Trichuris suis* (*T. suis*) show encouraging results [5], and larger clinical trials are currently in progress to fully confirm the efficacy of helminth treatment of MS. To avoid the obvious inconvenience of using living parasites, recent genome and transcriptome analysis of *T. suis* [6] have helped to pave the way to identify the parasite products with immunomodulatory capacities. In line with this approach, we have recently shown that soluble products of *T. suis* (TsSP) were able to ameliorate clinical parameters in experimental autoimmune encephalomyelitis (EAE), a well-established animal model for MS [7].

To date, the underlying protective mechanisms of helminth products are being revealed and include the induction of regulatory responses in the host [4], probably via the modulation of dendritic cells (DCs), which are key regulatory players of the adaptive immune response [8]. Our previous work showed that TsSP induce Th2 responses via DCs, whereas the induction of Th1 and Th17 responses by TsSP-primed human DCs is strongly reduced [7, 9]. Beside affecting adaptive immunity, we have recently reported that TsSP suppresses pro-inflammatory responses in monocyte-derived macrophages [10], which are key players in MS pathogenesis, as these cells are responsible for axonal loss and neurodegeneration [11]. In turn, these findings suggest that the observed beneficial effects of TsSP in MS patients may also be explained by a direct effect on innate immunity, and this hypothesis will be addressed in the current study. Key players of the human innate immune response are monocytes, which constitute around 10 % of the total leukocyte pool and are found in the blood, bone marrow and spleen. They originate in the bone marrow from hematopoietic stem cells [12] and are divided into subsets based on the surface expression of CD14 and CD16 [13]. The most prevalent monocyte subset in the blood consists of classical monocytes which display high CD14 levels and low CD16 levels (CD14^++^CD16^−^) and additionally express the chemokine receptor CCR2 [14]. Other subsets include CD16-expressing monocytes, which can be further divided in two subpopulations: CD14^+^CD16^+^ (intermediate) and CD14^dim^CD16^++^ (non-classical), which both express high levels of the chemokine receptor CX_3_CR1 [15]. Of these CD16-expressing monocytes, the non-classical cells are regarded as patrolling cells, as they adhere and migrate along the luminal surface of endothelial cells that line small blood vessels, similar to the mouse GR1^low^ monocyte population [16]. Genetic deletion and/or inhibitory antibody studies have shown that besides CX_3_CR1 [17], the patrolling behaviour of monocytes is also dependent on the integrin Lymphocyte Function-associated Antigen 1 (LFA-1;[16]), which binds to intercellular cell adhesion molecule-1 (ICAM-1) on endothelial cells [18]. In MS, the majority of monocytes display a classical (inflammatory) phenotype based on high CD40, CD86, HLA-DR, CD64 and CCR2 expression [19] and active MS lesions are dominated by monocyte-derived macrophages that have entered the CNS by traversing the blood–brain barrier (BBB). Therefore, research is needed to identify molecules and mechanisms that can skew these inflammatory monocytes into anti-inflammatory [16] and wound healing [20] patrolling cells that exhibit reduced transendothelial migration capacity, and thereby provide novel ways to combat disease progression.

As monocytes belong to the first responders to helminths as well as their secreted molecules and are regarded as crucial players in various auto-immune diseases, we hypothesized that these innate effector cells are prime targets for the helminths to exert their immunomodulatory effects. In the current study, we have investigated the effects of TsSP on human monocytes and report that TsSP potently affect the classical monocyte population by inducing a shift from classical to non-classical cells with reduced CCR2 expression and eliciting a differential pro- and anti-inflammatory cytokine response. TsSP-treated cells show a patrolling phenotype and display reduced monocyte adhesion and transendothelial migration capacities in an in vitro model of the BBB. Mechanistically, we identified the mannose receptor (MR) as the dominant TsSP-interacting monocytic receptor and revealed that protein kinase C (PKC), and in particular PKCδ signals downstream upon TsSP treatment. Overall, these data illustrate a potent anti-inflammatory effect of TsSP on human monocytes and thereby provide further mechanistic insight into the therapeutic potential of these helminth compounds in auto-immune diseases like MS.

## Materials and Methods

### TsSP and reagents

Soluble products of *Trichuris suis* (TsSP) were prepared as described previously [9, 10]. A limulus amebocyte lysate assay (Thermo scientific, USA) was used to determine endotoxin levels in TsSP, which appeared to be similar to background levels, thereby excluding LPS contamination. Cleavage of TsSP peptide chains (using α-chymotrypsin (CT, Sigma, USA) and oxidation of glycan moieties (using sodium periodate (PI; 10 mM, Sigma) was performed as described previously [9]. We used blocking antibodies for the human mannose receptor (MR, CD206, BD Pharmingen, USA) or Dectin-2 (Clone 545943, R&D Systems, USA). Recombinant human TNF-α was obtained from Invitrogen (Carlsbad, CA, USA). To study the involvement of PKC or Rho GTPases, we used the panPKC inhibitor Bisindolylmaleimide I (GF109203, Enzo Life Sciences, the Netherlands) as well as the Rac1-GEF inhibitor NSC-23766 (Bio-Techne, Abingdon, UK) and the Rho kinase inhibitor Y27632 (Sigma-Aldrich, Steinheim, Germany).

### Monocyte isolation

Peripheral blood mononuclear cells (PBMCs) were isolated using Ficoll density gradient (Lymphoprep™, Axis- Shield, Oslo, Norway) from buffy coats obtained from healthy donors (Sanquin Blood Bank, Amsterdam, the Netherlands) or patients who signed informed consent based on principles outlined in the Declaration of Helsinki. The studies were approved by the local ethics committee of Sanquin Blood Supply, Medical University of Vienna, Vienna, Austria and Comitè de Protection des Personnes Sud-EST IV, France. Patients' characteristics including details about *PRKCD* gene mutations have been described before [21, 22]. Monocyte isolation was performed by gradient centrifugation on Percoll (Amersham Biosciences, USA) according to manufacturer’s protocol. To increase monocyte purity, we used anti-CD14 magnetic beads (Miltenyi Biotec, Germany) with a MACS® MultiStand and LS Column by passing 3 ml of MACS buffer (2 mM EDTA and 0.1 % FCS in PBS) according to the manufacturers’ protocol. Monocyte purity (based on CD68 expression) was >90 % as assessed by flow cytometry performed (FACSCalibur™ using CELLQuest™ software (BD Biosciences) and monocytes were cultured in RPMI 1640 medium supplemented with 10 % heat-inactivated fetal calf serum (FCS), 2 mM L-glutamine, 100 U/ml penicillin and 100 μg/ml streptomycin (all obtained from Gibco-BRL Life Technologies, Breda, the Netherlands).

### Flow cytometry

Freshly isolated monocytes were cultured in the presence or absence of TsSP (40 μg/ml) for 16 h and subsequently harvested. Next, monocytes were washed twice with PBS and labelled for 1 h at 4 °C with primary antibody (Table 1) diluted in PBS containing 0.1 % BSA. Cells were washed twice in PBS and incubated for 1 h (4 °C) with fluorescent labelled secondary antibodies diluted in PBS/0,5 % BSA (FACS buffer), washed twice and resuspended in FACS buffer prior to FACS analysis. We used either four or eight colour flow cytometry. Four colour flow cytometry (FACSCalibur, Becton Dickinson, Belgium) was used in combination with Cell Quest software (Becton Dickinson) and FlowJo software version 9.4.0 for Microsoft (Tree Star, San Carlos, CA) to analyse expression of markers. For eight colour flow cytometry, we used a Cyan ADP High Performance Research Flow Cytometer (Beckman Coulter) and results were analyzed with Summit Software v4.3. Single stained cells were used to compensate for spectral overlap. Fluorescence Minus One (FMO) stained cells were used to set boundaries between positively and negatively stained cells. Sytox Blue dead cell stain (Molecular Probes, the Netherlands) was used to discriminate between live and dead cells.

**Table 1:** Antibodies used for FACS analysis

Antibody	Clone	Manufacturer
CD14-APC	MEM-15	Immunotools, Germany
CD16-FITC	LNK16	Immunotools, Germany
CCR2-biotin	48607	R&D systems, USA
CX_3_CR1	ab8021	Abcam, the Netherlands
LFA-1	L7	Dept. of Molecular Cell Biology and Immunology, Amsterdam, the Netherlands
LFA-1 (affinity)	L16	Dept. of Molecular Cell Biology and Immunology, Amsterdam, the Netherlands
LFA-1 (avidity)	Kim127	Kind gift from MK Robinson (UK)
CD206 (MR)	19.2	BD Pharmingen, USA

### Cytokine assays

The production of pro- and anti-inflammatory cytokines was assessed by enzyme-linked immunosorbent assay (ELISA) in cell-free supernatant samples using the Human Inflammatory 5-Plex Panel (Invitrogen, USA). This multiplex bead assay was performed according to the user manual supplied by Invitrogen. Samples were measured by Luminex® 200TM (Bio-Rad, California, USA) and analysed using Bio-plex ManagerTM 6.0 software.

### DHR assay

Reactive oxygen species (ROS) production of monocytes was measured using dihydrorhodamine (DHR), which reacts with ROS in a peroxidase-like reaction to yield fluorescent rhodamine 123 [23]. After culturing of monocytes in the presence or absence of TsSP (40 μg/ml) for 16 h, cells were rinsed twice with RPMI, incubated for 30 min at 37 ° C with 0.5 μM DHR (Sigma Aldrich, Germany) in RPMI medium. After that, cells were rinsed twice with PBS/0.1 % BSA, transferred to FACS tubes and analysed on a FACSCalibur (see above) with excitation at 488 nm and the emitted fluorescence collected at 525 nm.

### RNA isolation and quantitative real-time PCR

Messenger RNA (mRNA) was isolated using a mRNA Capture kit (Roche, Switzerland), and subsequently transcribed into cDNA using the Reverse Transcription System kit (Promega, USA), as described previously [24]. Quantitative real-time PCR was performed with the SYBR Green method as previously described [24]. Oligonucleotides were designed using Primer Express 2.0 (Applied Biosystems, USA) computer software. All primer sequences are listed in Table 2 and expression levels of transcripts obtained with real-time PCR were normalized to *GAPDH* expression levels.

**Table 2:** Primers used for RT-PCR

Gene	Forward (5’–3’)	Reverse (5’–3’)
GAPDH	CCATGTTCGTCATGGGTGTG	GGTGCTAAGCAGTTGGTGGTG
SOCS1	TGAACTCGCACCTCCTACCTCT	CAACCCCTGGTTTGTGCAA
IL-10	CGCTGTCATCGATTTCTTCCCT	AGGCATTCTTCACCTGCTCCAC
TGF-β	ACTATTGCTTCAGCTCCACGGA	AGTCAATGTACAGCTGCCGCA
TNF-α	GCCCATGTTGTAGCAAACCCT	ATGAGGTACAGGCCCTCTGATG
IL-6	AATTCGGTACATCCTCGACGG	GTTTGTTTTCTGCCAGTGCCT
CD206 (MR)	GTCTTGGGCCACAGGTGAA	AAGGCGTTTGGATAGCCACA

### Western blotting

Freshly isolated monocytes were cultured in the presence or absence of TsSP (40 μg/ml) for 30 min. Cell lysates were prepared using NP-40 lysis buffer (1 % Nonidet P-40, 10 % glycerol, 100 mM NaCl_2_, 10 mM MgCl_2_, 50 mM Tris, pH 7.4) mixed with protease and phosphatase inhibitors (Halt protease and phosphatase inhibitor cocktail, Thermo Fisher Scientific, Rockford, IL, USA). Prior to immunoblotting, lysates were boiled 10 min in Laemmli sample buffer containing 1 % β-mercaptoethanol. Equal sample volumes were subjected to 10 % sodium dodecyl sulfate–PAGE and transferred to nitrocellulose membranes (Schleicher & Schuell, Dassel, Germany). Membranes were washed with TSM/0,05 % Tween and blocked in TSM/0,05 % Tween/10 % roti-block (Techmate, Milton Keynes, UK, #A151.4). The following primary antibodies (in TSM/0,05 % Tween/5 % BSA) were used: PhosphoPKC (Cell signaling, Beverly, MA, USA, #9371S) and GAPDH (Santa Cruz Biotechnology, Heidelberg, Germany, #sc-32233) as a loading control. Followed by incubation with polyclonal goat anti-rabbit HRP (Dako, Heverlee, Belgium, #p0448) and polyclonal rabbit anti-mouse HRP (Dako, Heverlee, Belgium, #p0161). Proteins were detected using SuperSignal West Pico Chemiluminescent Substrate (Thermo Scientific, USA), in an EpiChemi II Darkroom (UPV Laboratory Products). Bands were quantified using Image J.

### Live cell imaging for assessment of monocyte motility

Real-time video recordings of monocytes were performed with an inverted phase-contrast microscope (Olympus, IX81-ZDC, Suffolk, U.K.) housed in a humidified, 5 % CO_2_ gassed, temperature-controlled (37 °C) chamber. For this, 3 × 10^5^ freshly isolated monocytes were applied in each well of an IBIDI slide in the presence or absence of TsSP (40 μg/ml), and randomly selected fields were recorded for 240 min. Pictures were taken every 3 min with an Olympus ColorView II camera (Olympus Nederland BV). Recordings were analyzed using CELL F trackIT software (Olympus Soft Imaging Solutions, Münster, Germany).

### Monocyte adhesion and migration assays

The immortalized human brain endothelial cell line hCMEC/D3, which preserves the key features of brain endothelium, was cultured as described [25]. Monocyte migration of primary human monocytes across confluent monolayers of hCMEC/D3 cells was studied with time-lapse video microscopy as described previously [26]. Freshly isolated monocytes were cultured in the presence or absence of TsSP (40 μg/ml) for 16 h and subsequently washed and added (7.5 × 10^5^ cells/ml) to hCMEC/D3 cells, and the number of migrated monocytes were counted after 4 h. For monocyte adhesion experiments, the TsSP-treated or untreated monocytes were fluorescently labelled with 0.5 μM BCECF-AM (Molecular Probes) for 15 min at 37 °C. Labelled monocytes (1 × 10^6^ cells/ml) were added to confluent monolayers of hCMEC/D3 cells and allowed to adhere for 30 min at 37 °C and 5 % CO_2_. Nonadherent cells were removed by gentle washing with prewarmed medium. Adhered cells were lysed with 0.1 M NaOH, and fluorescence intensity was determined (Fluostar 32, BMG; excitation 485 nm, emission 535 nm). The number of adherent monocytes was calculated using a calibration curve.

### Statistical analysis

All data were analyzed statistically by means of analysis of variance (ANOVA) and Student-*t* test. Statistical significance was defined as *p < 0.05, ** *p* < 0.01, *** *p* < 0.001.

## Results

### TsSP induce a shift from classical to non-classical human monocytes

**Fig. 1:**
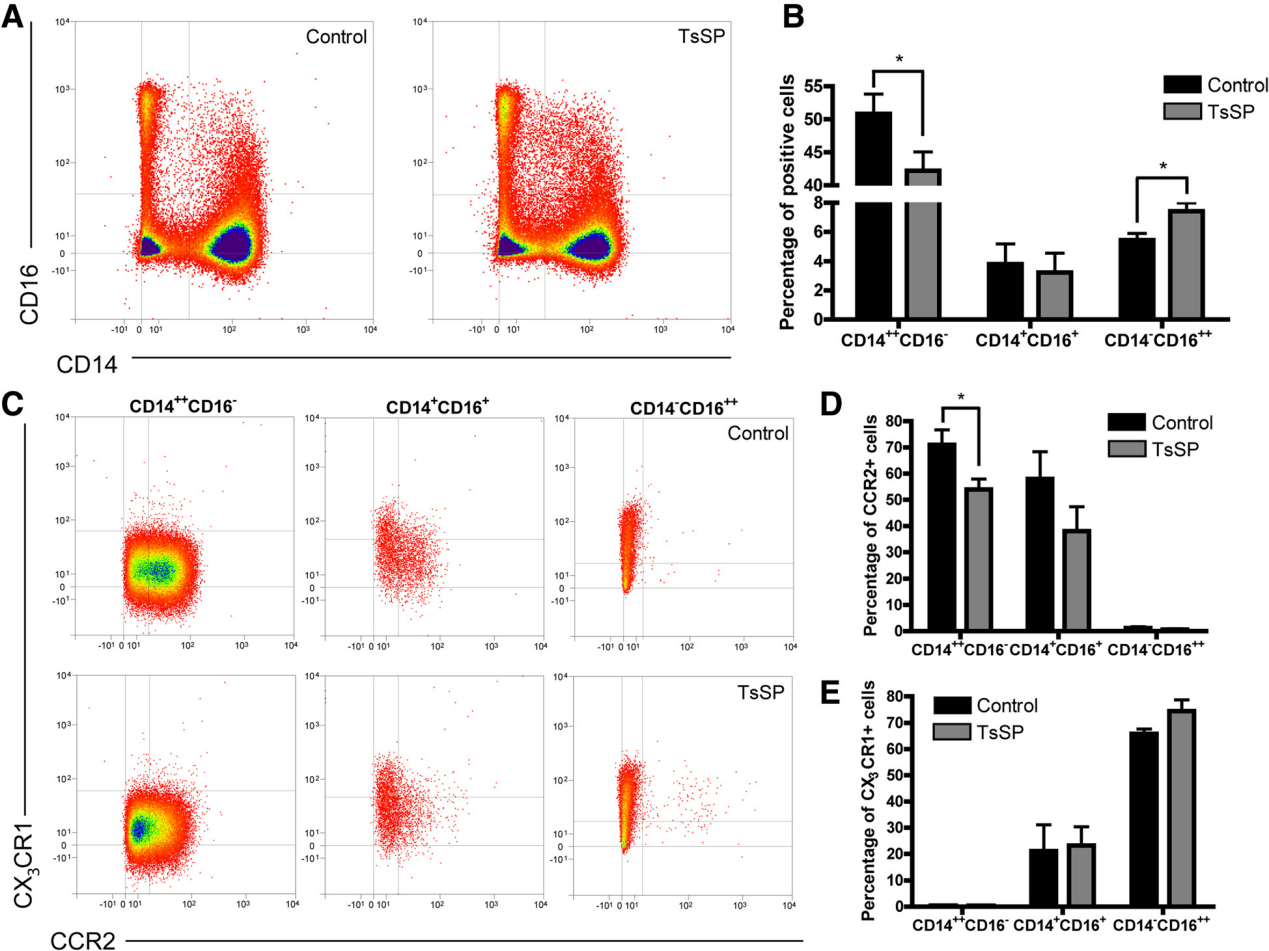
TsSP induce non-classical human monocytes. Primary human monocytes were cultured for 16 h in the presence or absence of TsSP (40 μg/ml) and subsequently analysed by flow cytometry for classical and non-classical monocyte markers like (**a** and **b**) CD14 and CD16 as well as (**c-e**) CCR2 and CX_3_CR1 on live gated cells. Experiments were performed in triplicate using cells derived from 5 different human donors and the results are presented by FACS plots (**a** and **c**) or the mean percentage of positive cells +/− SEM (**b, d** and **e**). *p < 0.05 as determined by Students *t* test

To identify the pathways by which *T. suis* soluble products (TsSP) affect the innate immune system, we first investigated whether TsSP causes changes in the phenotype of human blood monocytes. As shown in Fig. 1a and c, these circulating cells can be divided into two subpopulations; a large population of inflammatory CD14 expressing cells (CD14^++^CD16^−^, classical monocytes) with high CCR2 expression, and a small population of anti-inflammatory CD16 expressing cells (CD14^dim^CD16^++^, non-classical monocytes and a CD14^+^CD16^+^ intermediate population) with high CX_3_CR1 expression. Interestingly, TsSP treatment of monocytes results in a decreased percentage of classical CD14^++^CD16^−^ monocytes and an increased percentage of CD14^dim^CD16^++^ non-classical monocytes (Fig. 1b). Moreover, TsSP treatment significantly reduces the proportion of CCR2-positive cells in classical monocytes and slightly but not significantly induces the proportion of CX_3_CR1-positive cells in non-classical monocytes (Fig. 1d, e). These results indicate that TsSP predominantly affect classical monocytes, by inducing a shift from classical to non-classical cells.

**Fig. 2:**
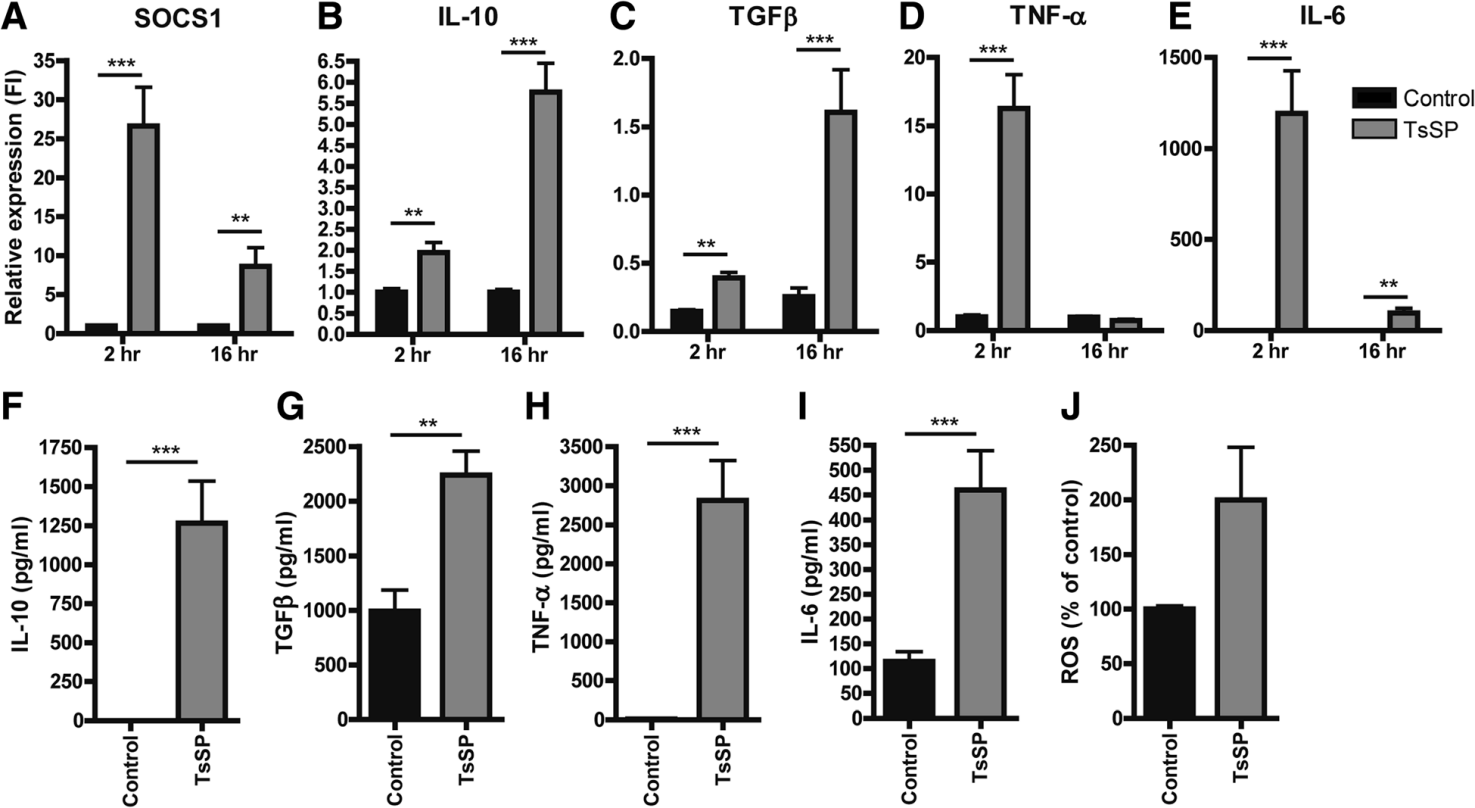
TsSP induce pro- and anti-inflammatory responses in human monocytes. Primary human monocytes were cultured for 2 or 16 h in the presence or absence of TsSP (40 μg/ml) after which various pro- and anti-inflammatory mediators were determined by (**a-e**) real-time quantitative PCR (qRT-PCR) and presented as relative expression (FI: fold induction) compared to *GAPDH*, by (**f-i**) enzyme-linked immunosorbent assay in cell supernatants (16 h) or by (**j**) dihydrorhodamine (DHR) flow cytometric assay to determine ROS (16 h). Experiments were performed in triplicate using cells derived from 5 different human donors and results are presented as the mean +/− SEM. **p < 0.01, ***p < 0.001 as determined by Students *t* test

To further investigate these phenotypic alterations, we analysed the effects of TsSP on gene expression levels of various anti-inflammatory markers including SOCS1, IL-10 and TGFβ, as well as pro-inflammatory cytokines like TNF-α and IL-6 in purified monocytes. As shown in Fig. 2a-e, TsSP treatment significantly induces gene expression of both pro- and anti-inflammatory markers. Next, the levels of secreted IL-10, TGFβ, TNF-α and IL-6 were determined, as well as the production of reactive oxygen species (ROS), a typical marker of inflammatory monocytes [16]. In line with the transcriptional results (Fig. 2a-e), TsSP significantly enhances the secretion (Fig. 2f-i) and production (Fig. 2j) of these pro- and anti-inflammatory mediators. Of note, the induction of anti-inflammatory genes occurs predominantly at later time points (16 h) compared to the induction of pro-inflammatory genes (2 h), thereby distinguishing the acute inflammatory response to the pathogen from the apparent secondary anti-inflammatory response. Together, these results suggest that TsSP potently affect the classical monocyte population, by lowering their CCR2 expression. In turn, this may lead to a shift into non-classical cells and concomitantly elicit a differential cytokine response.

### TsSP induce motility behaviour in human monocytes

**Fig. 3:**
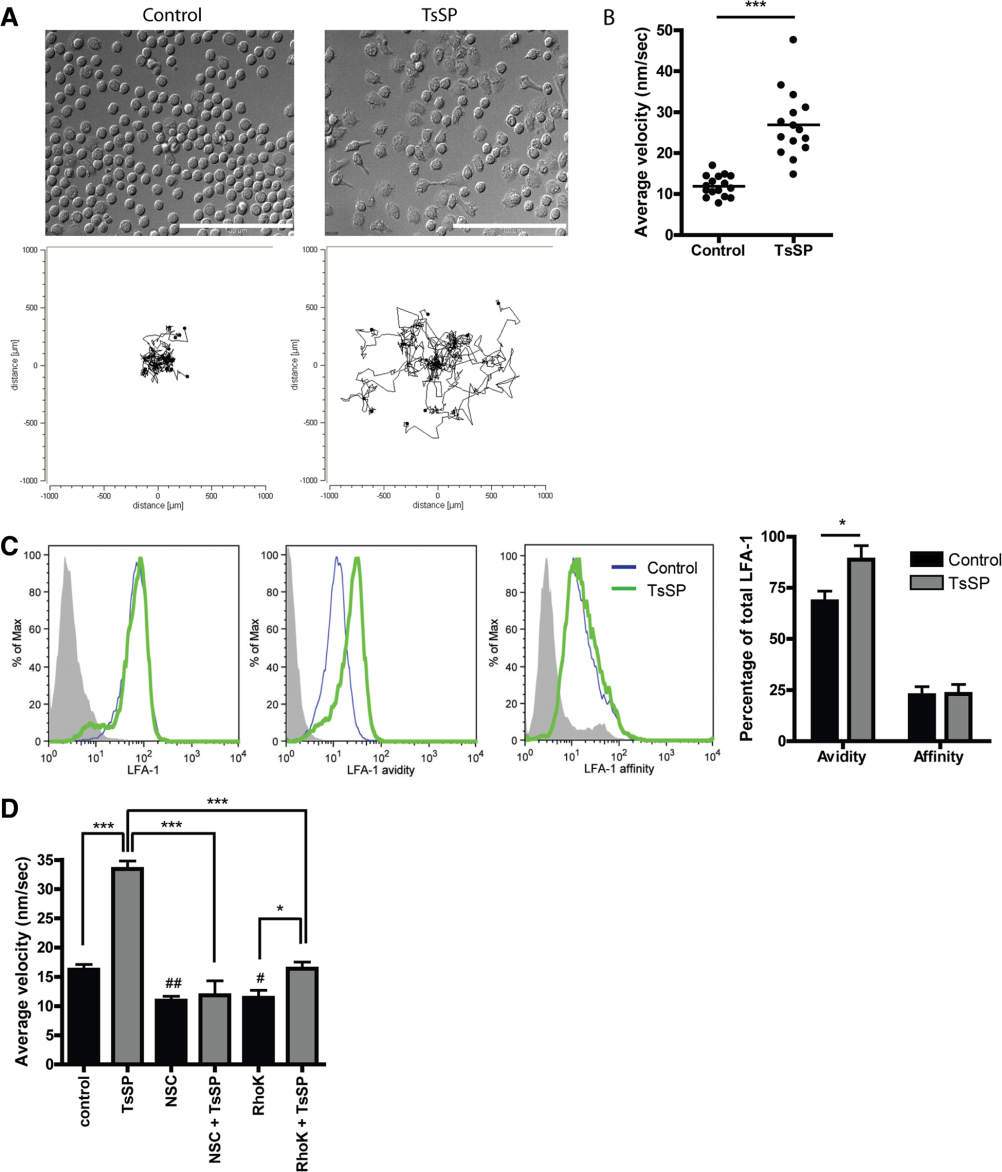
TsSP induce patrolling motility behaviour in human monocytes and increase LFA-1 avidity. Addition of TsSP (40 μg/ml) to primary human monocytes instantly induces chemokine-independent cell motility resembling patrolling monocytes as determined by live cell imaging. **a** Snap shot of control (left) or TsSP-treated monocytes (right). **b** Quantification of 2-dimensional motility (10 h) as well as travelled distance by using Image J. **c** Flow cytometry analysis and quantification of LFA-1 (total, avidity and affinity) expression levels on human monocytes in the presence or absence of TsSP (40 μg/ml). **d** Monocyte motility assays were performed in the presence or absence of NSC-23766 (Rac inhibitor) or Y27632 (Rho Kinase inhibitor). Experiments were performed in triplicate using cells derived from 8 (**a** and **b**) or 5 (**c** and **d**) different human donors and the results are presented as the mean +/− SEM. *p < 0.05, ***p < 0.001 or ^#^ *p* < 0.05, ^##^ *p* < 0.01 (compared to control) as determined by ANOVA and Students *t* test

It was previously shown that human non-classical monocytes have a distinct (patrolling) motility behaviour in vivo, thereby representing the human homologue of the murine patrolling Gr1^low^ monocytes with anti-inflammatory capacities [16]. To test the effect of TsSP on the motility of human monocytes, we performed live cell imaging experiments. Notably, TsSP*-*treated monocytes display extensive crawling behaviour (Electronic supplementary material (Additional file 1: video 1 (control) and Additional file 1: video 2 (TsSP-treated cells)), showing both a higher undirected motility and more distance travelled compared to untreated cells (Fig. 3a,b). This patrolling behaviour was previously shown to be dependent on CX_3_CR1 [17] as well as lymphocyte function-associated antigen-1 (LFA-1, [16]). We did not observe a significant increase in CX_3_CR1 expression upon TsSP treatment (Fig. 1e). However, by using specific antibodies to determine LFA-1 affinity (Kim127, [27]) and avidity (L16, [28]), we found that TsSP treatment specifically increases LFA-1 avidity in human monocytes, whereas total LFA-1 as well as LFA-1 affinity levels remained unaltered (Fig. 3c). In general, cell motility is regulated by the activity of small Rho GTPases like Rho, Rac and Cdc42, which have a direct effect on the actin cytoskeleton [29]. To investigate whether the TsSP-induced monocyte motility is dependent on Rho GTPases, we performed live cell imaging experiments in the presence or absence of specific Rho GTPase inhibitors like NSC-23766 (Rac1) and Y27632 (Rho kinase inhibitor). As shown in Fig. 3d, blocking these Rho GTPases significantly limits the TsSP-induced patrolling behaviour, indicating that these GTPases play a key role in the TsSP-induced signalling pathway. Overall, these results indicate that TsSP induces a patrolling behaviour in monocytes in a Rac/Rho GTPase-dependent manner.

### TsSP-induced motility of human monocytes is glycan-dependent

**Fig. 4:**
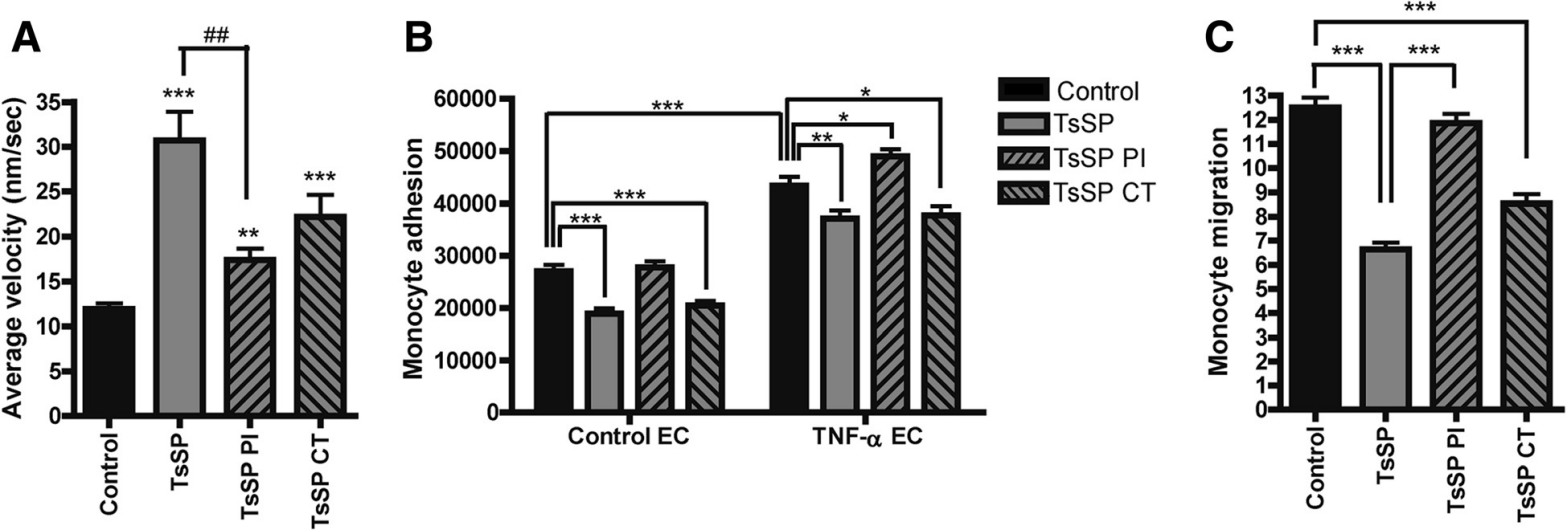
TsSP-induced functional effects on monocytes are glycan-dependent. Primary human monocytes were treated with TsSP, periodate (PI)- or chymotrypsin (CT)-treated TsSP (all 40 μg/ml) after which the cell motility was instantly visualized by (**a**) live cell imaging (10 h) and subsequently quantified by using Image J. Monocytes that were incubated with TsSP or PI/CT-TsSP for 16 h were used to study (**b**) monocyte adhesion to brain endothelial cells as well as (**c**) monocyte transendothelial migration as studied by time-lapse video microscopy. These experiments were performed on control (**b**) or TNF-α-treated endothelial cells (**b** and **c**). Experiments were performed in triplicate using cells from 8 (**a**) or 5 (**b** and **c**) different human donors and the results are presented as the mean +/− SEM. *p < 0.05, ** or ^##^ *p* < 0.01, ***p < 0.001 as determined by ANOVA and Students *t* test

We have previously shown that TsSP can downregulate inflammatory responses in DCs in a glycan-dependent manner [9]. To test whether the effects we observed on monocytes after TsSP treatment are glycan- and/or protein-mediated effects, we used periodate (PI) to oxidize glycan structures on TsSP or chymotrypsin (CT) to disrupt protein structures. As shown in Fig. 4a, PI treatment of TsSP caused a profound loss of the TsSP-induced patrolling behaviour of monocytes, whereas CT treatment caused a small but not significant reduction. These results illustrate an important role for TsSP-glycans in the induction of patrolling monocytes.

### TsSP-treatment of human monocytes impedes their BBB transendothelial migration

A crucial hallmark in the pathogenesis of MS is the central nervous system (CNS) entry of leukocytes, which ultimately causes severe parenchymal tissue destruction. To enter the CNS, monocytes adhere to and finally transmigrate across brain endothelial cells (ECs) of the blood–brain barrier (BBB). To test whether TsSP treatment of monocytes affects these processes, we analysed monocyte adhesion in vitro, as well as their transendothelial migration using human brain ECs [25]. To mimic the inflammatory conditions as seen in MS, we treated the ECs with the inflammatory cytokine TNF-α, thereby inducing the expression of adhesion molecules like ICAM-1 and VCAM-1 [30], resulting in enhanced monocyte adhesion to brain EC (Fig. 4b). Importantly, TsSP treatment of monocytes significantly reduced their adhesion capacity, both under control and inflammatory conditions (Fig. 4b). In this regard, TsSP glycans also play a role, since PI treatment of TsSP completely abolished the induced reduction of monocyte adhesion (Fig. 4b). Furthermore, TsSP treatment of monocytes profoundly reduced their transendothelial migration capacity across TNF-α-treated EC by 50 % in a glycan-dependent manner (Fig. 4c). Together, these results indicate that periodate-sensitive moieties within TsSP modulate monocyte behaviour, by inducing patrolling cells and hampering their adhesion and transmigration capacity.

### TsSP interacts with the mannose receptor on human monocytes

**Fig. 5:**
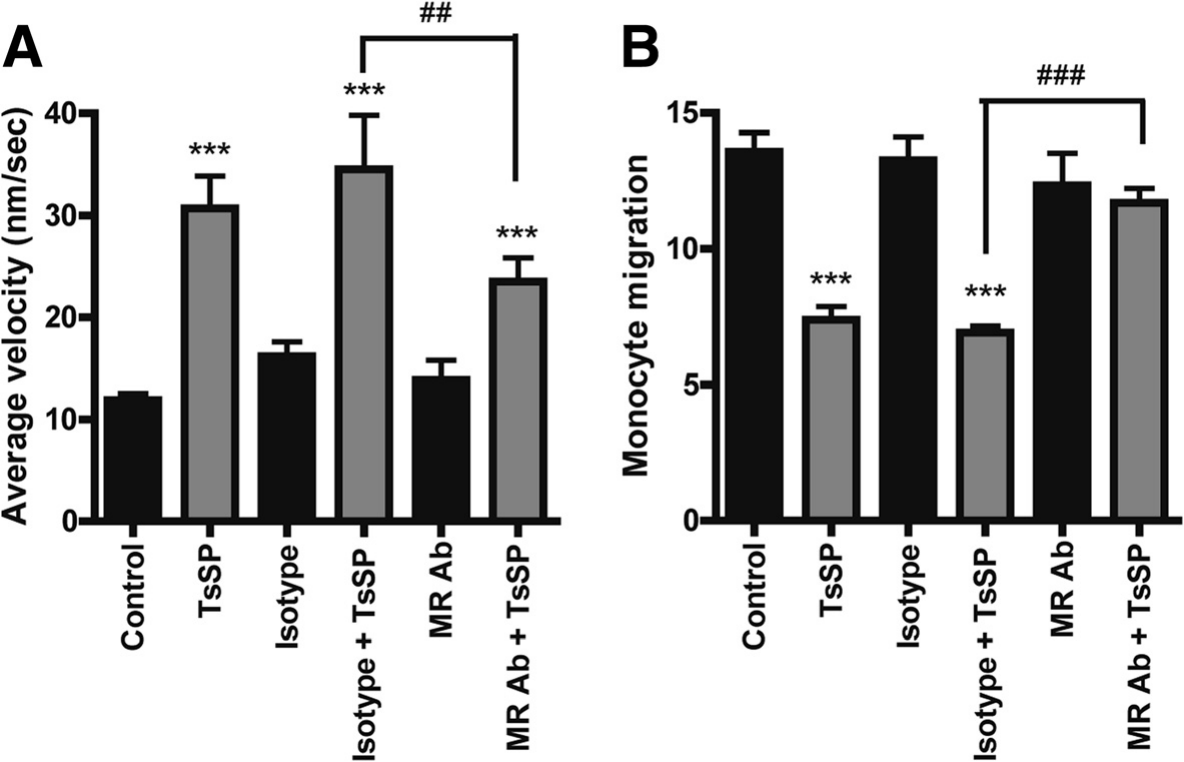
TsSP interacts with the MR on human monocytes. The effect of blocking antibodies for the mannose receptor (MR, CD206) or its isotype control (both 10 μg/ml) on (**a**) the TsSP-induced patrolling phenotype or (**b**) transendothelial migration capacity was tested and subsequently quantified. Experiments were performed in triplicate using cells derived from 8 (**a**) or 5 (**b**) different human donors and the results are presented as the mean +/− SEM. ** or ^##^ *p* < 0.01, ***p < 0.001 as determined by ANOVA and Students *t* test

The contribution of periodate-sensitive TsSP moieties in the modulation of monocyte function suggests that glycans on TsSP may be recognized by glycan-binding receptors such as C-type lectins (CLRs), leading to an altered monocyte behaviour. We have recently shown that DCs can bind to TsSP via the mannose receptor (MR, CD206) and that TsSP are strongly bound by ConA, suggesting the presence of oligo-mannose-type glycans on TsSP [9]. Next to the MR, the TsSP glycans may also be recognized by other monocytic CLRs with mannose recognizing potential like Dectin-2 [31]. To evaluate this possibility, we first analyzed the expression of such CLRs on monocytes, which show that both the MR and Dectin-2 are expressed and that MR expression is induced upon TsSP treatment (Additional file 2). Next, we evaluated the involvement of the MR and Dectin-2 in modulating monocyte behaviour. Interestingly, treatment of monocytes with TsSP in the presence of a MR blocking antibody significantly inhibited the TsSP-induced patrolling behaviour of monocytes (Fig. 5a) and rescued the TsSP-induced reduction in monocyte trans-endothelial migration (Fig. 5b). In contrast, blocking of Dectin-2 did not affect monocyte patrolling behaviour and monocyte transendothelial migration (Additional file 3), suggesting that TsSP predominantly exerts its effect on human monocytes via the MR.

### TsSP induce PKC signaling downstream of the MR

**Fig. 6:**
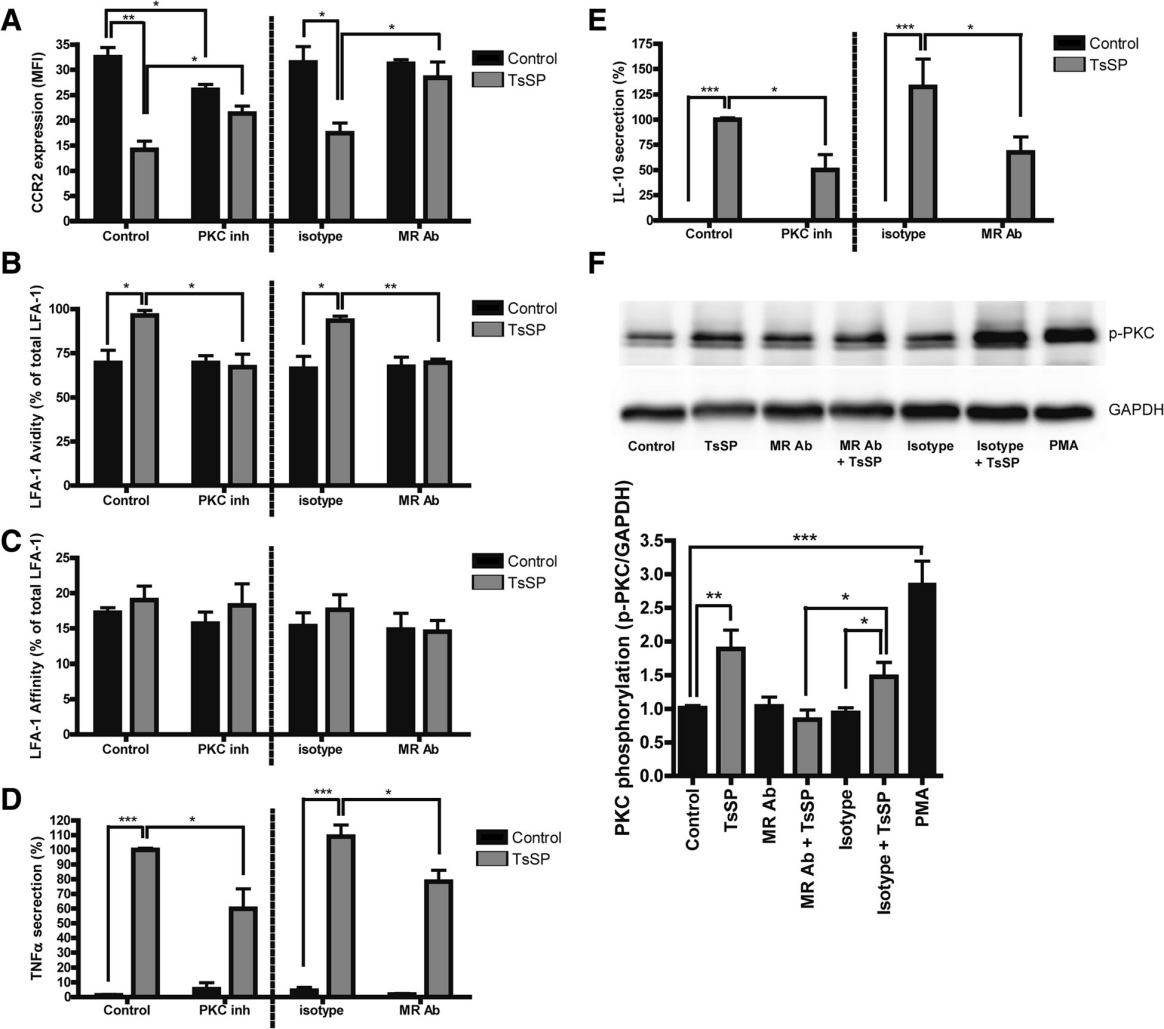
PKC signals downstream upon TsSP-MR interaction. Quantification of flow cytometry analysis of the expression levels of (**a**) CCR2, (**b**) LFA-1 avidity and (**c**) LFA-1 affinity on human monocytes. These cells were cultured in the presence or absence of TsSP (40 μg/ml, 16 h), the PKC inhibitor Bisindolylmaleimide I (GF109203, 2 μM) and blocking antibodies for the MR or its isotype control (both 10 μg/ml). (**b**) and (**c**) are expressed as the percentage of avidity/affinity compared to total LFA-1. Cell supernatants were used to determine the levels of (**d**) TNF-α and (**e**) IL-10 secretion by enzyme-linked immunosorbent assay analysis. **f** PKC activation was determined by Western Blotting using phosphoPKC antibodies compared to *GAPDH* expression and images were subsequently quantified using ImageJ. Experiments were performed in triplicate using cells derived from 4 different human donors and the results are presented as the mean (**a-c**); the mean percentage (**d, e**) or the mean PKC phosphorylation +/− SEM. *p < 0.05, **p < 0.01, ***p < 0.001 as determined by ANOVA and Students *t* test

The MR has a short cytoplasmic tail lacking ITIM or ITAM signalling motifs [32] and its downstream signalling pathway remains largely elusive. As CCR2 expression and LFA-1 activation can be regulated by PKC [33, 34], we hypothesized that the observed effects of TsSP on CCR2 expression and LFA-1 avidity (Figs. 1c and 3c) were MR and PKC-dependent. Indeed, as shown in Fig. 6a, the TsSP-induced downregulation of CCR2 was significantly rescued in the presence of the panPKC inhibitor bisindolylmaleimide I (GF109203) as well as in the presence of blocking antibodies to the MR. Moreover, the TsSP-induced LFA-1 avidity was completely abolished after blocking the MR as well as in the presence of GF109203 (Fig. 6b), whereas LFA-1 affinity remained unaffected (Fig. 6c). Finally, the TsSP-differential cytokine profile as evidenced by TNF-α and IL-10 secretion was significantly reduced after blocking the MR as well as in the presence of GF109203 (Fig. 6d, e). Together, these results indicate that TsSP interact with the MR on monocytes and affect their phenotype via PKC.

To provide evidence that TsSP directly activates PKC via the MR, we performed Western Blotting using phospho-PKC antibodies. As shown in Fig. 6f, TsSP treatment significantly induces PKC phosphorylation, to a similar level as the well-known PKC activator phorbol 12-myristate 13-acetate (PMA). Importantly, this TsSP-induced PKC phosphorylation was completely abrogated in the presence of blocking antibodies to the MR, thereby providing direct evidence that TsSP induce a PKC-dependent signalling pathway via the MR in human monocytes.

### TsSP predominantly exerts its effect on human monocytes via PKCδ

**Fig. 7:**
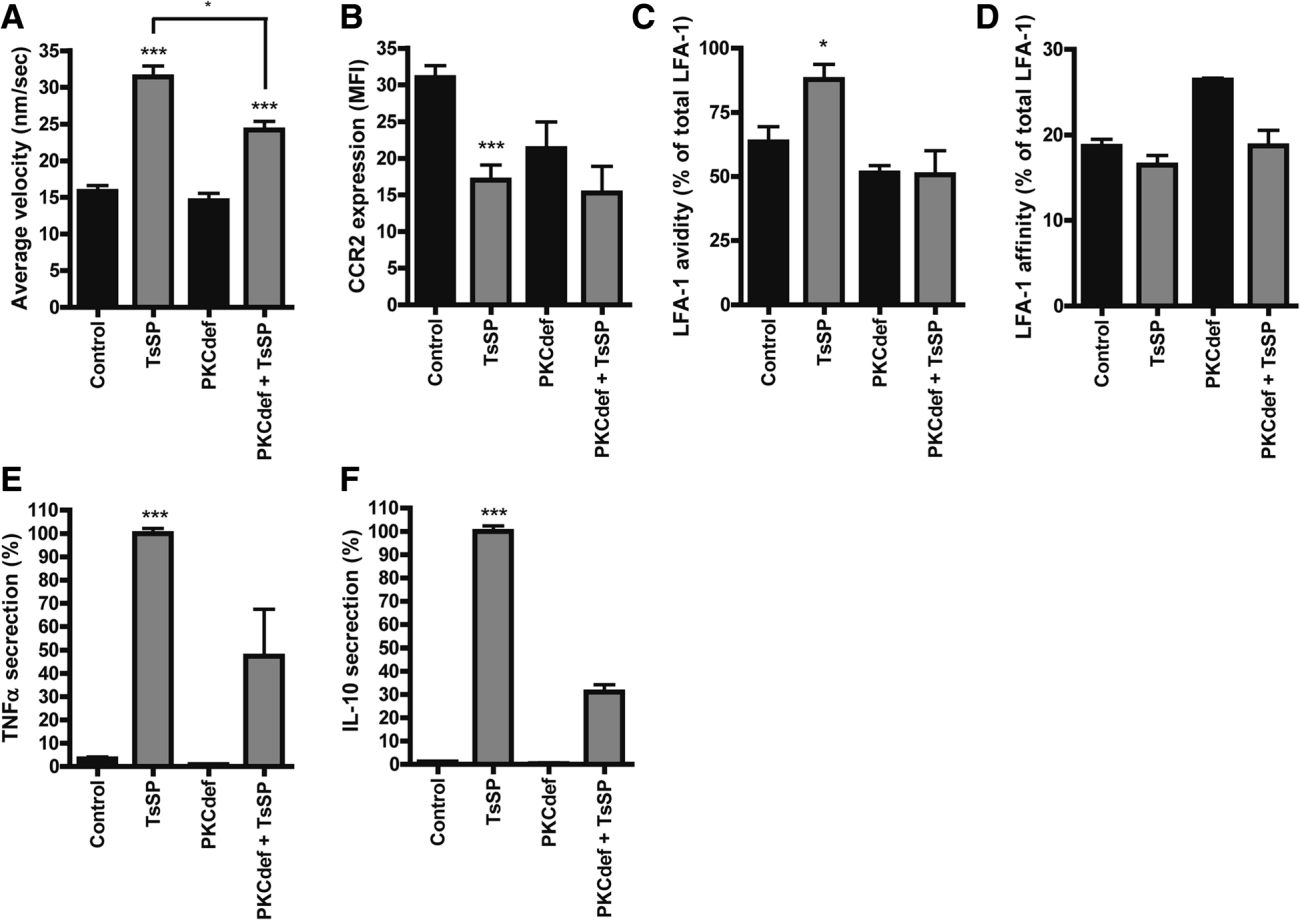
TsSP modulates monocytes via PKCδ signaling. Control or PKCδ-deficient human monocytes were treated with TsSP (40 μg/ml) after which (**a**) the patrolling cell phenotype was visualized by live cell imaging (10 h) and subsequently quantified by using Image J and the expression levels of (**b**) CCR2, (**c**) LFA-1 avidity and (**d**) LFA-1 affinity were analyzed and quantified using flow cytometry. Cell supernatants were used to determine the levels of (**e**) TNF-α and (**f**) IL-10 secretion by enzyme-linked immunosorbent assay analysis. Experiments were performed in triplicate using 3 human control donors (**a-f**) and 3 (**a-d**) or 2 (**e, f**) PKCδ-deficient donors and results are presented as the mean (**a, b**) or the mean percentage (C-F) +/− SEM. *p < 0.05, ***p < 0.001 as determined by ANOVA and Students *t* test

The PKC family consists of classical (PKCα, βI, βII, and γ), novel (PKCδ, ε, θ, and η), and atypical (ζ and ι/λ) members, and in mice it was recently suggested that PKCδ may signal downstream of the MR [35]. However, data in a human setting are lacking and research on PKCδ is complicated by the fact that specific inhibitors are currently unavailable [36]. To circumvent this and to assess the role of PKCδ in the TsSP-induced monocyte modulation, we isolated and analysed monocytes from patients with extremely rare mutations in the *PRKCD* gene [21, 22] that either lack PKCδ or have a considerably reduced expression in monocytes-derived macrophages [36]. Importantly, the TsSP-induced patrolling behaviour in control cells was significantly impaired in PKCδ-deficient monocytes (Fig. 7a). PKCδ-deficient monocytes showed a trend towards decreased CCR2 expression, which remained unaltered after TsSP treatment (Fig. 7b). Lack of PKCδ completely abolished the TsSP-induced LFA-1 avidity (Fig. 7c), whereas LFA-1 affinity remained unaffected (Fig. 7d). Moreover, the TsSP-induced TNF-α and IL-10 secretion was severely impaired in PKCδ-deficient monocytes (Fig. 7e, f). Together, these findings indicate that TsSP predominantly exerts its effect on human monocytes via PKCδ signalling.

## Discussion

Helminths have the capacity to survive in their host by modulating the immune response towards a tolerant or non-inflammatory response. Unravelling their mechanism of action may provide a key to combat various inflammatory diseases. Indeed, recent clinical trials in MS patients show beneficial results of administration of probiotic helminths, and their protective mechanism of action has been largely attributed to the induction of regulatory immune responses via adaptive immunity. However, as innate immune cells like monocytes belong to the first responders to parasite infections and are regarded as crucial players in MS pathogenesis, we here evaluated the effect of the porcine nematode *Trichuris suis* soluble products (TsSP) on monocyte phenotype and function. We demonstrate that TsSP affect classical monocytes, by inducing a shift from classical to non-classical cells with decreased adhesion to and migration across a human in vitro blood–brain barrier (BBB). Mechanistically, we show that TsSP interacts with the mannose receptor (MR, CD206) and we identified PKC and in particular PKCδ as the main signalling molecule downstream of the MR responsible for the observed altered monocyte functions. Overall, this study provides mechanistic insights into the effect of TsSP on the innate immune response, which further unravels their protective effect to combat various inflammatory diseases, including MS.

During infection, *T. suis* helminths reside in the highly vascularised abdominal regions were they constantly secrete soluble products and dampen host immune responses [37]. We have previously shown that secreted products of *T. suis* can reduce the barrier integrity of intestinal epithelial cells [38], thereby facilitating the entry of helminth products into the lamina propria as well as the vasculature. Herein, circulating monocytes encounter these products and in the current study we have mimicked this initial encounter. We here show that TsSP treatment affects classical monocytes by reducing their CCR2 expression and inducing a shift into non-classical monocytes. At the functional level, we demonstrate that TsSP potently reduce monocyte adhesion to and transmigration across the BBB in vitro. Noteworthy, the TsSP induced effect on monocyte adhesion seems contradicting to the observed TsSP-induced LFA-1 avidity changes in human monocytes. However, it must be noted that firm adhesion requires both LFA-1 avidity and affinity conformational changes [39], and we therefore propose that the observed TsSP-induced LFA-1 avidity alone is not sufficient to enhance the adhesion of monocytes to brain endothelial cells. A key pathological hallmark of MS lesion formation is the central nervous system (CNS) entry of monocyte-derived macrophages across the vasculature, as these infiltrated cells are associated with axonal loss, astrogliosis and neurodegeneration [11]. We have previously shown that CCL2, the ligand for CCR2 is highly expressed by brain endothelial cells from MS patients [30] and several other studies have indicated that the CCL2/CCR2 axis is crucial for the entry of monocytes to the site of inflammation [40]. Moreover, mice that lack CCR2 are resistant to the induction of EAE [41, 42], thereby revealing the relevance for the CCL2/CCR2 axis for neuro-inflammation. Importantly, CCR2-deficient leukocytes show impairments in their adhesion and migration capacity across endothelial cells [43]. Therefore, the observed TsSP effects on monocyte adhesion and transmigration may be largely attributed to a loss of CCR2 expression.

Compared to control, MS patient monocytes display a pronounced inflammatory profile, including high expression of CD40, CD86, CD64 and CCR2 [19]. The identification of molecules and mechanisms that can skew these inflammatory cells into anti-inflammatory [16] and wound healing [20] patrolling cells may provide novel ways to combat disease progression. In the current study, we provide first evidence that TsSP may exert such a shift in monocyte phenotype. Interestingly, in vivo studies have indicated that the murine equivalent (GR1^low^ monocytes [16] or type II monocytes) can significantly reduce clinical signs of EAE [44, 45], further illustrating that a shift in the monocyte population from inflammatory to anti-inflammatory cells can be regarded as a promising therapeutic avenue. In that way, TsSP may act in a similar way as a currently approved MS drug glatiramer acetate (Copaxone), which promotes the development of anti-inflammatory monocytes in vivo [45]. Next to that, it is tempting to speculate that patrolling monocytes may resemble the precursors for alternatively activated macrophages (AAM or M2 macrophages) that are required for proper tissue restoration after an inflammatory event [46]. Although this differentiation has been shown for murine Ly6C^−^ monocytes [47], this transition in a human setting remains to be shown.

Helminths and their soluble products are glycosylated, and the glycans of several helminth species have been shown to be essential for the induction of Th2 responses [48]. We here extend these initial findings and show that periodate-sensitive glycan moieties on TsSP are required for the observed altered monocyte functions. These glycan moieties can be recognized by specific glycan-binding proteins such as C-type lectins (CLRs), and in the current study we identified the MR as the TsSP-interacting human monocytic receptor. The MR is a type-1 membrane protein with a single transmembrane domain and a cytoplasmic domain that mediates receptor internalisation and recycling [49]. It can bind a wide variety of exogenous and endogenous molecules and therefore is thought to mediate both homeostatic and immune processes [50]. However, as MR-deficient animals do not display enhanced susceptibility to pathogens that contain MR-ligands [51, 52], its function in host defence is not fully understood. It has been shown that *Trichuris muris*, which is the murine equivalent of *T. suis,* was able to interact with the MR on macrophages. However, experiments with MR-deficient animals showed that this interaction was not needed for the expulsion of the parasite [53]. The diverse secretome of helminths [54] may interact with various cellular receptors, but based on our findings we propose that the anti-inflammatory effect on human monocytes is largely MR-dependent. It has been show that the MR is involved in the recognition and uptake of various pathogens [50] and further research is warranted to reveal which glycan-ligands of TsSP interact with the MR and how these molecules are further processed within the cell. Interestingly, we observed that upon MR-TsSP interaction, monocytes display increased motility and crawling behaviour, similar to the murine patrolling Gr1^−^ monocytes with anti-inflammatory capacities [16]. Although it has previously been suggested that the MR may be involved in cell motility [50], these are the first data to provide actual evidence for this in a human setting.

To date, the downstream signalling pathway of the MR remains largely elusive, and in the current manuscript we unravelled that PKC, and in particular PKCδ signals downstream of the MR upon TsSP interaction, thereby regulating LFA-1 activation, CCR2 expression and cytokine secretion. PKC isoforms are important intracellular signalling molecules involved in cell differentiation, migration, proliferation and activation [55]. Our findings confirm previous observations that CCR2 expression and LFA-1 avidity are regulated by PKC [33, 34] but extend these findings to i) a human setting and ii) by the identification of the upstream cellular receptor (MR) as well as the identification of PKCδ-signaling in this regard. To date, the role of PKCδ in human monocytes has hardly been studied due the fact that specific PKCδ inhibitors are currently not available [36] as well as the general appreciation that other PKC isoforms (PKCα and PKCβ) are the more dominant members present in monocytes [56]. Importantly, there are only five patients worldwide known with a mutation in the *PRKCD* gene [21, 22]. In the current study, we used monocytes from three different PKCδ-deficient patients, which aided our studies in showing that TsSP exerts its effect on human monocytes predominantly via PKCδ signalling.

## Conclusion

In conclusion, we here show a potent anti-inflammatory effect of TsSP on human monocytes and identified the MR as the TsSP-interacting receptor as well as specific downstream signalling via PKC. In turn, this study provides first insight into the beneficial effect of TsSP on the innate immune system, which together with our recent described effects of TsSP on the adaptive immune responses [7, 9] provides comprehensive insight in their therapeutic potential to combat various inflammatory diseases, including MS. Further research is warranted to unravel the identity of the TsSP molecules with immunomodulating capacity, in order to more specifically dampen pathogenic immune responses under inflammatory conditions and subsequently avoiding the use of whole worms as a therapy.

## Additional files

Additional file 1:
**Video 1 and 2 are live cell imaging movies that show monocyte motility under control (video 1) or TsSP conditions (video 2).**
Additional file 2:
**shows MR and Dectin-2 protein (2a) and gene expression (2b) levels in human monocytes upon TsSP treatment.**
Additional file 3:
**shows the results from a monocyte motility assay (3a) and monocyte transendothelial migration assay (3b) to asses the role of Dectin-2 in the TsSP-induced monocyte functional alterations.**

## Abbreviations

7AAD: 7-Aminoactinomycin D
BBB: Blood brain barrier
CCL2: C-C chemokine ligand type 2
CCR2: C-C chemokine receptor type 2
CNS: Central nervous system
CT: Chymotrypsin
CLR: C type lectin receptor
CX_3_CR1: Fractalkine receptor
DC: Dendritic cell
EAE: Experimental autoimmune encephalomyelitis
EC: Endothelial cell
ICAM-1: Intercellular adhesion molecule 1
IL: Interleukin
LFA-1: Lymphocyte function-associated antigen 1
MR: Mannose receptor
MS: Multiple sclerosis
PI: Periodate
PKC: Protein kinase C
ROS: Reactive oxygen species
SOCS1: Suppressor of cytokine signaling 1
TGFβ: Transforming growth factor beta
TNF-α: Tumor necrosis factor alpha
TsSP: *Trichuris suis* soluble products
VCAM-1: Vascular cell adhesion protein 1
