# Supplementary figures and images for: Trichuris suis induces human non-classical patrolling monocytes via the mannose receptor and PKC: implications for multiple sclerosis
Source: Acta Neuropathol Commun. 2015 Jul 25;3:45. doi: 10.1186/s40478-015-0223-1 (PMC4513676; doi:10.1186/s40478-015-0223-1)

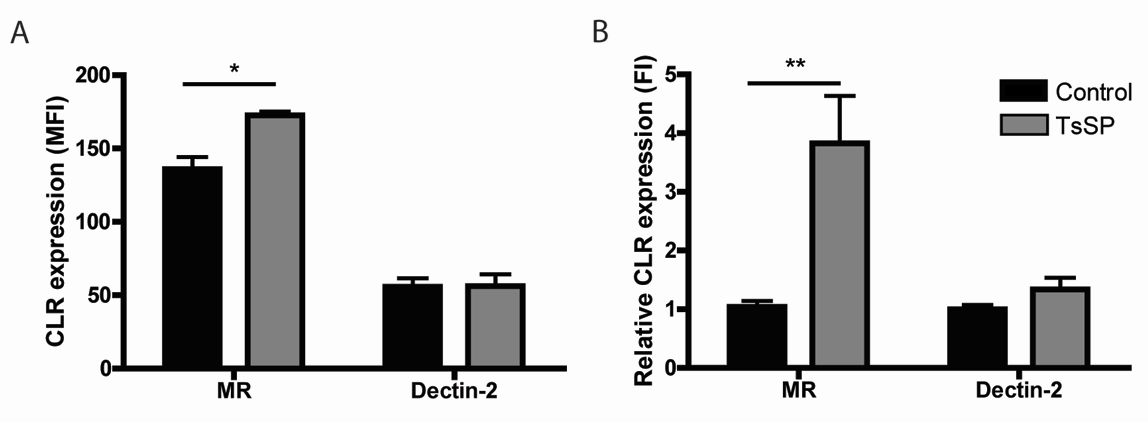

Supplement: Additional file 2: — shows MR and Dectin-2 protein (2a) and gene expression (2b) levels in human monocytes upon TsSP treatment. [file 40478_2015_223_MOESM2_ESM.tif]

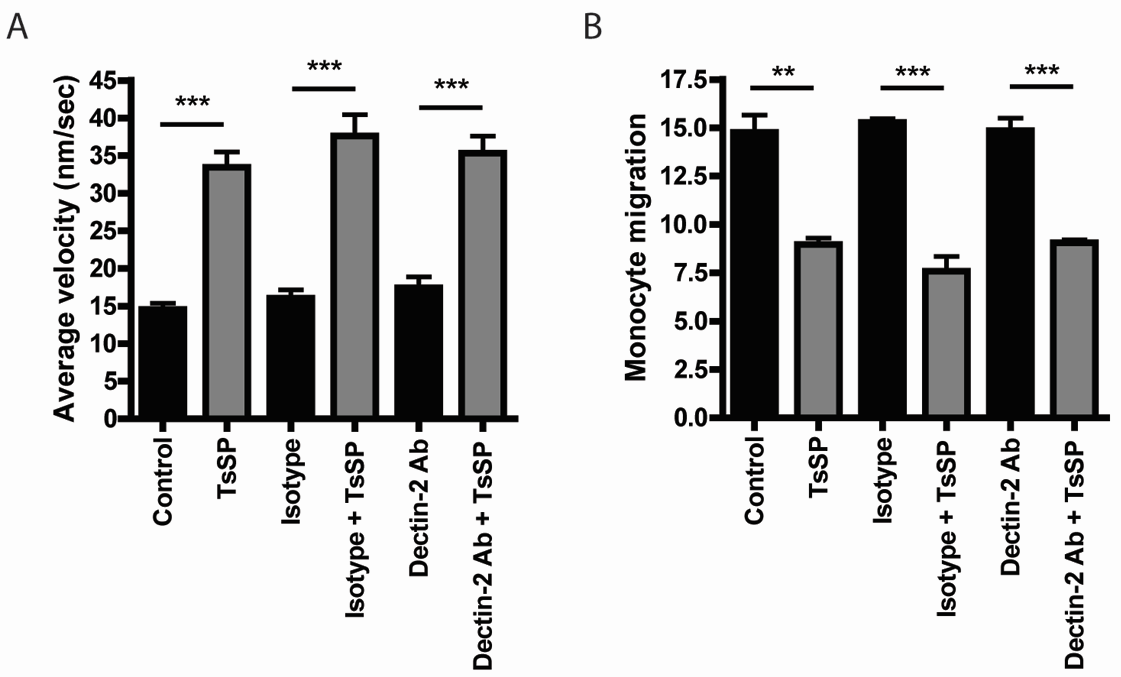

Supplement: Additional file 3: — shows the results from a monocyte motility assay (3a) and monocyte transendothelial migration assay (3b) to asses the role of Dectin-2 in the TsSP-induced monocyte functional alterations. [file 40478_2015_223_MOESM3_ESM.tif]
